# Labview Based ECG Patient Monitoring System for Cardiovascular Patient Using SMTP Technology

**DOI:** 10.1155/2015/701520

**Published:** 2015-12-10

**Authors:** Om Prakash Singh, Dawit Mekonnen, M. B. Malarvili

**Affiliations:** ^1^Biomedical Engineering, JiT, Jimma University, 378 Jimma, Ethiopia; ^2^Department of Biotechnology and Medical Engineering, Faculty of Bioscience and Medical Engineering, Universiti Teknologi Malaysia, 81310 Skudai, Johor, Malaysia

## Abstract

This paper leads to developing a Labview based ECG patient monitoring system for cardiovascular patient using Simple Mail Transfer Protocol technology. The designed device has been divided into three parts. First part is ECG amplifier circuit, built using instrumentation amplifier (AD620) followed by signal conditioning circuit with the operation amplifier (lm741). Secondly, the DAQ card is used to convert the analog signal into digital form for the further process. Furthermore, the data has been processed in Labview where the digital filter techniques have been implemented to remove the noise from the acquired signal. After processing, the algorithm was developed to calculate the heart rate and to analyze the arrhythmia condition. Finally, SMTP technology has been added in our work to make device more communicative and much more cost-effective solution in telemedicine technology which has been key-problem to realize the telediagnosis and monitoring of ECG signals. The technology also can be easily implemented over already existing Internet.

## 1. Introduction

**Figure 1:**
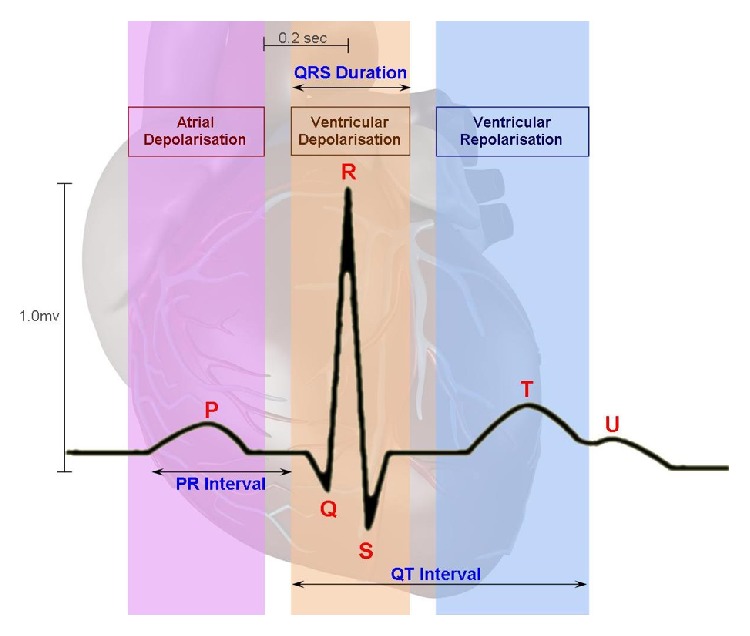
Peaks of ECG.

ECG is used to measure the rate and regularity of heartbeats, the presence of any damage to the heart, and the effects of drugs or devices used to regulate the heart (such as a pacemaker). Normally, the frequency range of ECG signal is 0.05–100 Hz and its dynamic range is 1–10 mV. The ECG signal as depicted in Figure 1 is characterized by five peaks and valleys labeled by the letters P, Q, R, S, and T. The performance of ECG analyzing system depends mainly on the accurate and reliable detection of the QRS complex, as well as T- and P-waves. The P-wave represents the activation of the upper chambers of the heart, the atria, while the QRS complex and T-wave represent the excitation of the ventricles or the lower chamber of the heart. The detection of the QRS complex is the most important task in automatic ECG signal analysis. Once the QRS complex has been identified a more detailed examination of ECG signal including the heart rate and the ST segment can be performed [1, 2].

Most of the modern 12-lead ECG monitoring systems are based on Einthoven's triangle, Wilson central terminal, and Goldberger augmented leads [3].

**Figure 2:**
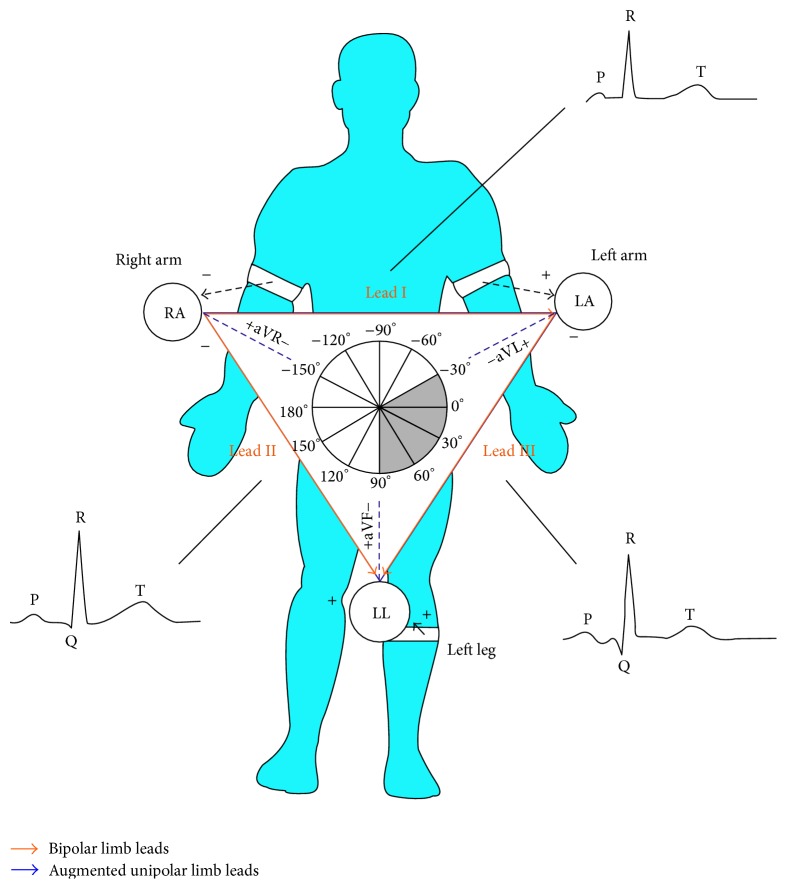
Electrode placement using a 3-wire cable.

The developed ECG device is implemented on the principle of Einthoven's triangle and used lead-II configuration, as it is known as a monitoring lead, given in Figure 2.

Based on above facts, there have been numerous attempts to develop medical systems similar to the work. Such efforts are primarily led by the academia but extending deeply into the industries. However, most research efforts have been focusing on either the vital sign monitoring aspect or the ECG feature extraction using standard databases both falling short of expectation. Having analyzed the existing solutions, this work vows to bridge the two major research efforts and bring out a more realizable product to directly benefit the consumers in the medical field.

This research work offers the following contributions to the produced system; foremost is the portable ECG monitoring platform based on a 3-lead system and a design under the NI DAQ card (6008). The ECG data was collected through the DAQ card to the PC/laptop and then transmitted to the end user (physician) through SMTP to analyze the patient condition.

## 2. Material and Method

**Figure 3:**
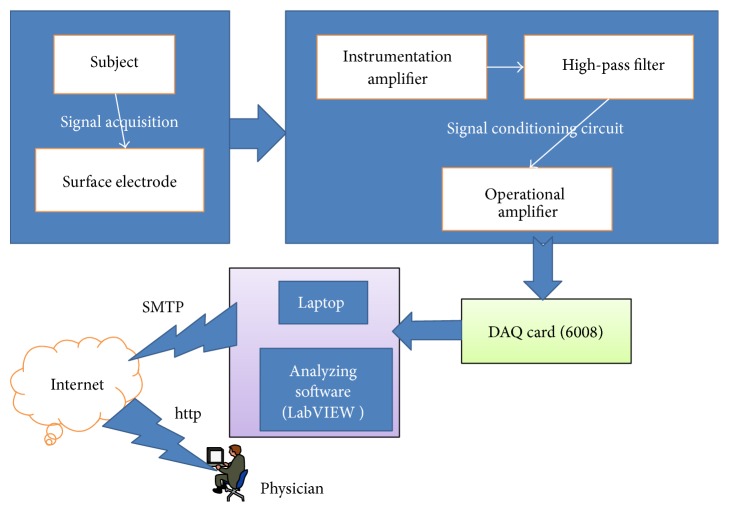
Block diagram of proposed ECG device.

The block diagram of the complete system is given in Figure 3. The complete design was divided into two parts: hardware and software.

The hardware part comprises instrumentation amplifier (AD620), some passive components, operational amplifier (LM741), DAQ card, and laptop whereas Labview is used as software. The software is used to exchange the data from analog to digital form, to perform the calculations, and to produce the ECG waveform onto the monitor. Each of the components in this block diagram is explained in detail in the following subsections [4].

### 2.1. Surface Electrode

The principle of the electrodes is to convert a physical parameter into an electrical output. The function of the transducer is to convert biological information into a quantifiable electrical signal. The transducer interface is provided using an electrode-electrolyte interface. The most preferable electrode is Ag/AgCl, as it reduces the impedance while using it and the gel is used for the proper contact in between the surface of the skin and electrode.

### 2.2. Signal Conditioning Circuit

After receiving raw ECG signal from the subject through electrode, it has to be processed in order to bring the signal in visible form and to limit the bandwidth of the signal. To do so, the instrumentation amplifier was used to amplify the tiny signal whereas passive and active components are used to design filter and to amplify it.

**Figure 4:**
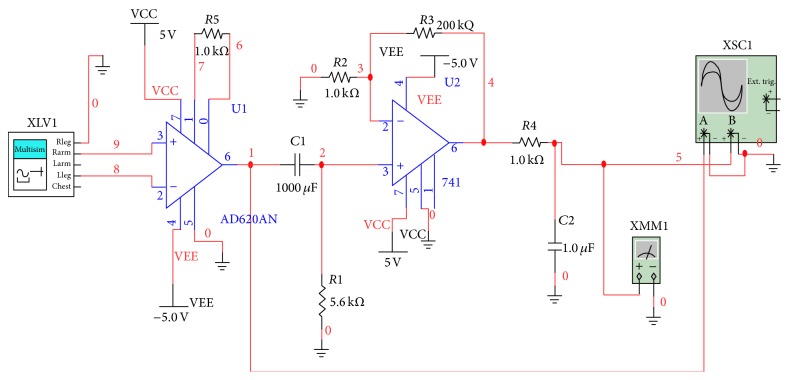
ECG signal acquisition and condition circuit.

Figure 4 illustrates the constructive circuit diagram of signal conditioning. The circuit has been designed and tested in multisims to get the appropriate output of signal.

#### 2.2.1. Instrumentation Amplifier

The voltage gain of the instrumentation amplifier is calculated using the following equation:

(1) G=1+49.4 kΩRg,G=1+49.4 kΩ1 kΩ=1+49.4=50.4.

#### 2.2.2. Operational Amplifier

The voltage gain of the operational amplifier is estimated using given formula as the used one is noninverting amplifier:

(2) G=1+R3R2,G=1+200 kΩ1 kΩ=1+200=201.

#### 2.2.3. High-Pass Filter

The output of the instrumentation amplifier is fed into the passive AC coupling with a cutoff frequency, as in (3), of 0.02 Hz such that high-pass filters

(3) fhigh-pass12πR1C1=12∗3.14∗5.6∗103∗1000∗10−6,fhigh-pass53.14∗56,fhigh-pass0.02 Hz.

#### 2.2.4. Low-Pass Filter

The second-stage amplified signal is fed into a low-pass filter with a cutoff frequency, as in (4), of 160 Hz for removing high frequency noise or movement artifacts:

(4) flow-pass12∗π∗R4∗C2=12∗3.14∗10∗103∗1∗10−6,flow-pass10006.28=159.2 Hz.

### 2.3. DAQ Card (6008)

The output of the signal conditioning circuit should be sent to the NI DAQ card for the conversion of signal from analog to digital as it has the inbuilt analog-to-digital converter.

**Figure 5:**
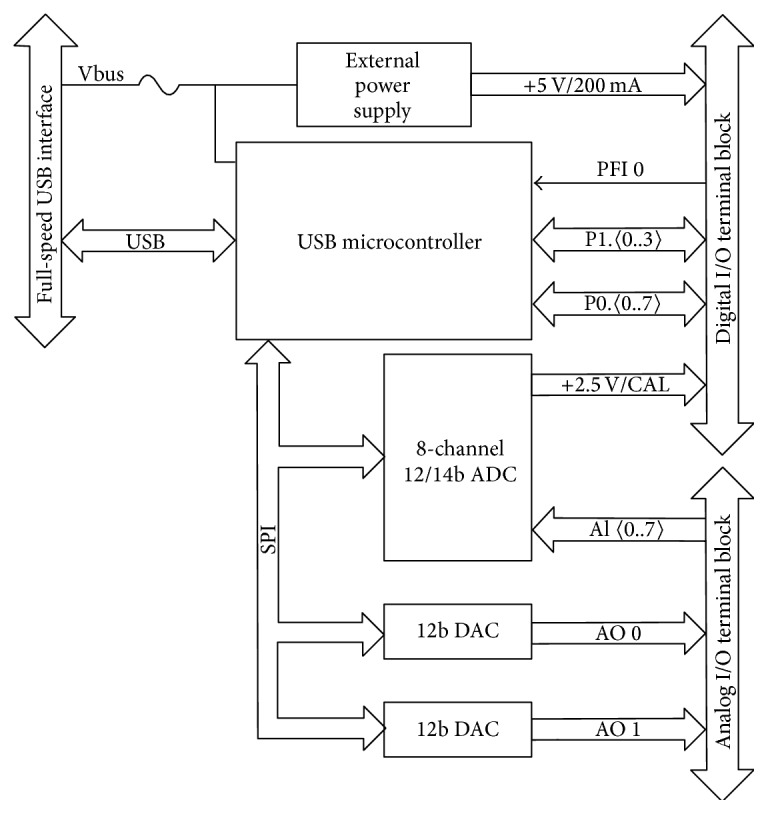
NI USB-6008 block diagram.

The given figure (Figure 5) gives the idea about the block diagram of NI USB-6008 which is a simple and low-cost multifunction I/O device from National Instruments [5]. It is used to digitize the amplified, filtered ECG signal. The NI USB-6008 card has 8 differential analog input channels and 12-bit analog-to-digital converter running at a sampling frequency up to 300 Hz, which can be increased up to 200 KHz. This satisfies the sampling requirements of the ECG signal.

### 2.4. Laboratory Virtual Instrument Engineering Workbench (Labview)

It is a graphically programmed computer language for real-time instrumentation. It is a software package developed to build programs with symbols (icons) rather than writing out lines and lines of programming text. It uses symbols, terminology, and formats that are familiar to technicians, scientists, and engineers. Labview is programmed to act as an interface, helping pieces of hardware “communicate” with each other. Moreover, Labview offers built-in libraries that allow the user to work over the Internet and use different programming formats and systems.

### 2.5. Simple Mail Transfer Protocol (SMTP)

**Figure 6:**
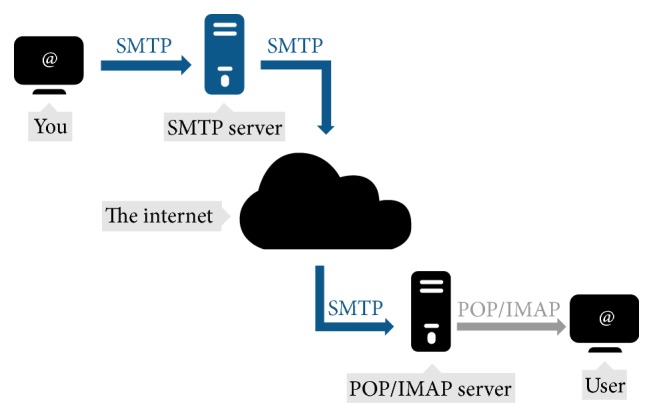
Simple Mail Transfer Protocol (refer to http://www.serversmtp.com/en/free-smtp-server).

Figure 6 shows the step of SMTP of how the data is processed throughout the different stages and transferred to client. It is a part of the application layer of the TCP/IP protocol. Using a process called “store and forward,” SMTP moves your e-mail on and across networks. It works closely with something called the Mail Transfer Agent (MTA) to send your communication to the right computer and e-mail inbox [http://support.aycontrol.com/].

## 3. Designing Strategies

**Figure 7:**
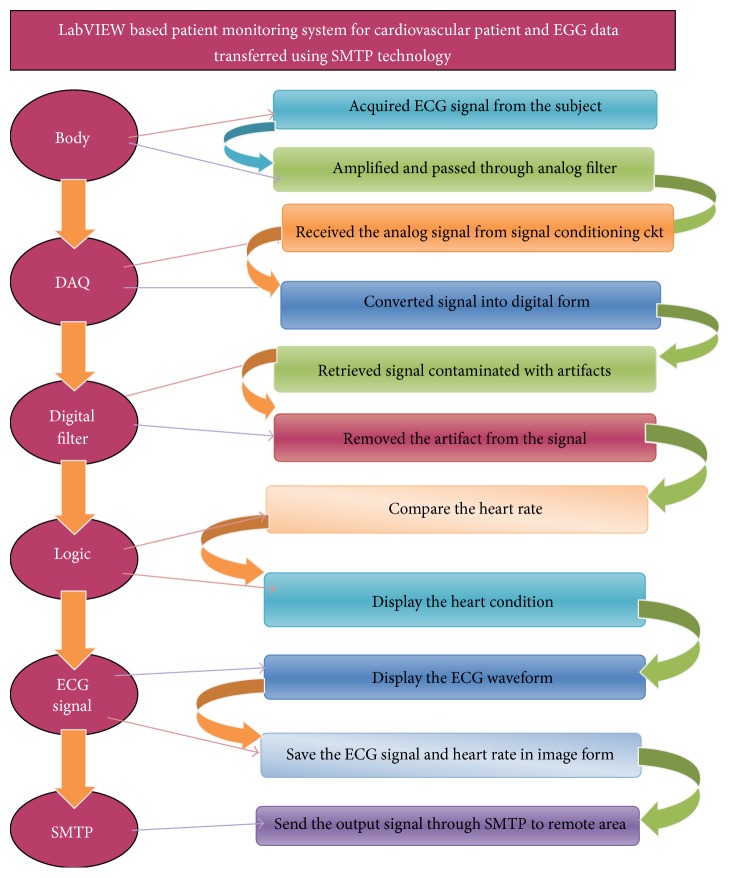
Designing strategy.

The approach that has been followed in the designing of device is included in Figure 7. There were several stages like finding the appropriate electrodes to acquire the signal from body of the subject and analog signal conditioning circuit includes filtering and amplifying stage, DAQ card, and digital signal processing and display system.

## 4. Designed Labview Interface

The ECG system that has been developed is used for the continuous acquisition and chart recording of single input channels. It allowed user to record and save buffered analog ECG data from one or more individuals, which is continuously acquired into a circular buffer at the same time in that data previously retrieved from the buffer is plotted [6–8]. A common reason to read data while the acquisition is in progress is to process and display the data in virtual-real time.

### 4.1. Retrieving Input Signal

The analog signal is acquired from the bread board and converted to the digital one through DAQ. Digital signal processing is done accurately as needed in order to produce high signal-to-noise ratio so that the heart diagnostic system is precise.

### 4.2. Digital Signal Processing

Here, the digital filter is done to remove the power line interferences and other artifacts available in the signal. The necessary data processing to display real-time ECG had been performed.

### 4.3. Threshold and Peak Detector

The threshold and peak indicators are implemented after processing. The purpose of these indicators is to provide some feedback to the user (i.e., physician) regarding the patient's average heart rate.

**Figure 8:**
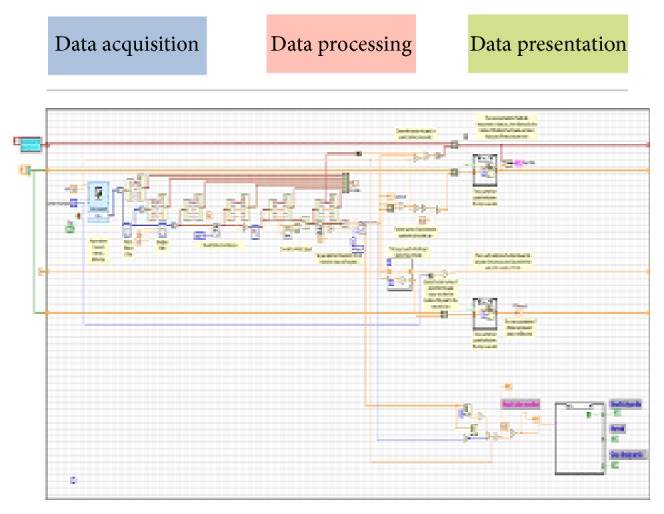
Acquired, processed, and calculated heart rate.

Figure 8 gives the idea about the complete ECG system developed in Labview. The first part shows how the data have been acquired using data acquisition card (6008); immediate in next step, the retrieved data has been processed by Band Pass Filter as well as notch filter to remove the artifacts from the signal [9, 10]. Furthermore, the algorithm is developed to detect the peak of the signal.

The following given equation is used to calculate the heart rate and to identify the arrhythmia condition [11, 12]:

(5) $$\begin{array}{c} \text{Heart Rate}=\frac{60}{t_{2}-t_{1}}, \end{array}$$

with *t*_1_ being occurrence of first R wave and *t*_2_ being occurrence of second R wave.

### 4.4. Display System Tool (Toshiba)

**Figure 9:**
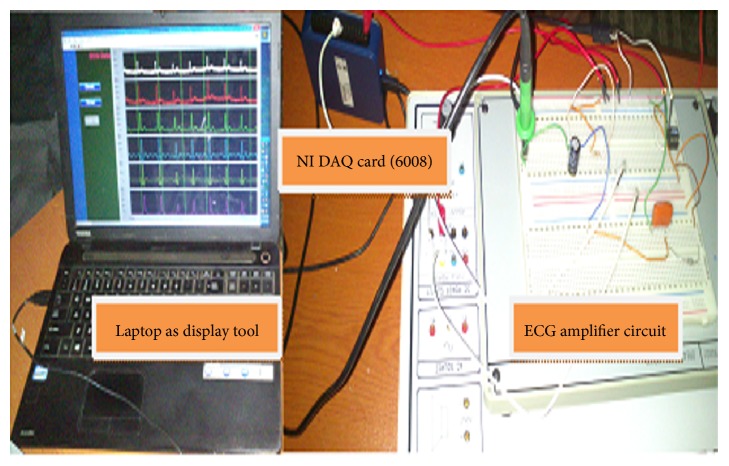
Proposed ECG device.

After processing raw ECG signal, the signal has been displayed onto the laptop as shown in Figure 9. The display too is compatible with the prerequisite of the Labview software.

### 4.5. SMTP (Simple Mail Transfer Protocol)

**Figure 10:**
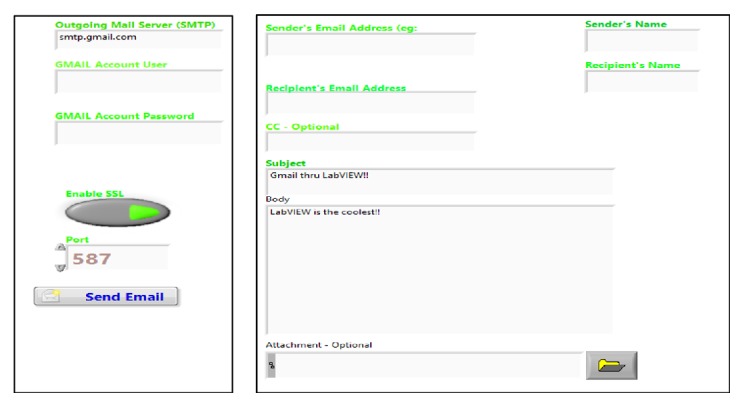
Front panel of SMTP.

**Figure 11:**
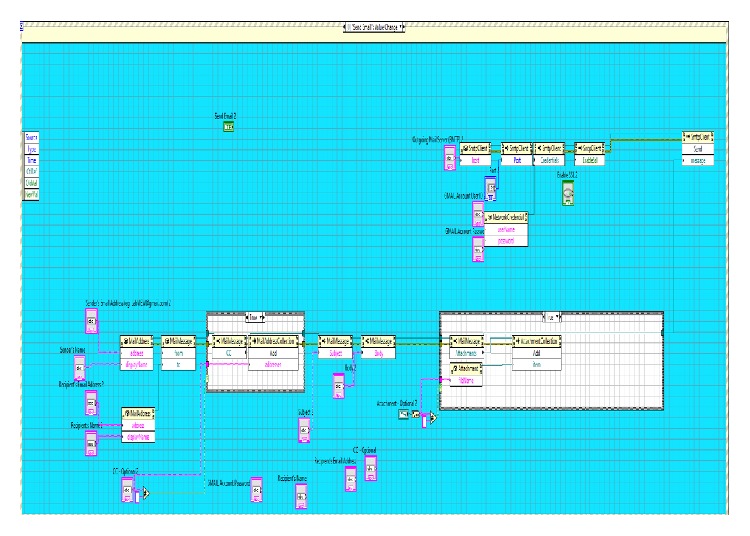
Block panel of SMTP.

Finally, the developed SMTP tool is used to send image file of acquired ECG signal through Email Express VI, shown in Figure 10 that represents the front panel and Figure 11 that represents the block panel developed using Labview which allows sending the data quickly through e-mails from Labview to a health care centre or a physician.

## 5. Result

**Figure 12:**
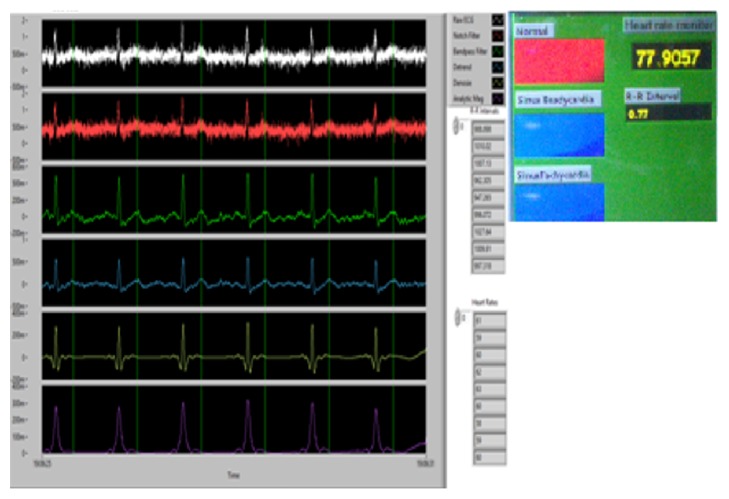
Acquired, processed ECG signal.

The given system is based on the principle of heart rate monitoring; it is able to produce the results shown in Figure 12, which show accurate ECG signals and the correct calculation of both a resting and an elevated heart rate. Initially, there was an issue with the system regarding doctor notification subsystem which has been short by validating the proposed concept by running the VI at home, which sent both text and e-mail to our “doctor” notifying them that there was a detected problem with the patient's heart. It brings the freedom for the physician as well as for the doctor to check up on patients hearts from time to time by seeing real-time waveforms as shown in Figure 12.

The proposed device is functioning well once all the hardware connected properly to meet the criteria for the proposed idea. As a whole, it is very reliable and portable as well as cost effective.

## 6. Discussion

Most of the work has been done based either on hardware or on software. In the case of hardware, to transmit the ECG data, transmitter and receiver had been used, which increases the cost of the device, whereas the developed one integrated with hardware along with software to transmit the data anywhere using SMTP technology. Thus, the complete device becomes more user friendly as well as cost effective.

## 7. Conclusion and Future Work

In this paper, the low-cost biomedical measurement system with the ability of storage in digital format as well as sending the data to the remote area has been presented. The hardware implementations using commercially available devices and the software written in Labview program for continuously monitoring ECG data have been described. The proposed measurement system is also capable of sending the data through SMTP to the physician or health care centre with no time. The proposed system could be modified by increasing the number of channels.
